# Three-step dissection with straight needle suturing for anvil placement in Natural Orifice Specimen Extraction Surgery (NOSES)

**DOI:** 10.1093/jscr/rjaf1008

**Published:** 2025-12-23

**Authors:** Haiyang Huang, Yonglong Kuang, Kaer Chen

**Affiliations:** Department of Gastrointestinal Surgery, Guangdong Taishan People's Hospital, 80 Huanbei Avenue, Taishan, Guangdong, 529200, China; Department of Gastrointestinal Surgery, Guangdong Taishan People's Hospital, 80 Huanbei Avenue, Taishan, Guangdong, 529200, China; Department of Gastrointestinal Surgery, Guangdong Taishan People's Hospital, 80 Huanbei Avenue, Taishan, Guangdong, 529200, China

**Keywords:** anvil placement, intracorporeal colorectal anastomosis, single-stapling technique, laparoscopic sigmoidectomy, Natural Orifice Specimen Extraction Surgery (NOSES), straight needle

## Abstract

This report outlines a modified technique for anvil placement in three-port single-stapling Natural Orifice Specimen Extraction Surgery (NOSES). A 72-year-old man underwent a procedure for sigmoid colon adenocarcinoma, with monitoring of operation time, blood loss, and complications; pathology confirmed the removal of a T3N0 tumor. This modification simplifies anvil placement and may improve NOSES adoption.

## Introduction

The double-stapling technique is the primary method for reconstructing the digestive tract in NOSES IV [1, 2]. However, the single-stapling technique, despite its challenges, may reduce complications [3–6] in rectal cancer surgery, encouraging experienced surgeons to use it for reconstruction [7]. The NOSES procedure eliminates the need for specimen removal through the abdominal wall and enhances reduced-port laparoscopic surgery for colorectal cancer [8]. Recent advances have allowed surgeons to perform procedures independently, leading to reduced-port NOSES and increased reports of single-operator three-port NOSES [9, 10]. However, operation times tend to be longer than in traditional NOSES, especially with narrow intestinal tracts, because placing the anvil effectively during intracorporeal anastomosis remains challenging. This highlights the need for efficient anvil positioning methods during colorectal anastomosis. This report introduces a simple three-step dissection technique with straight needle suturing for anvil placement.

## Case report

**Figure 1:**
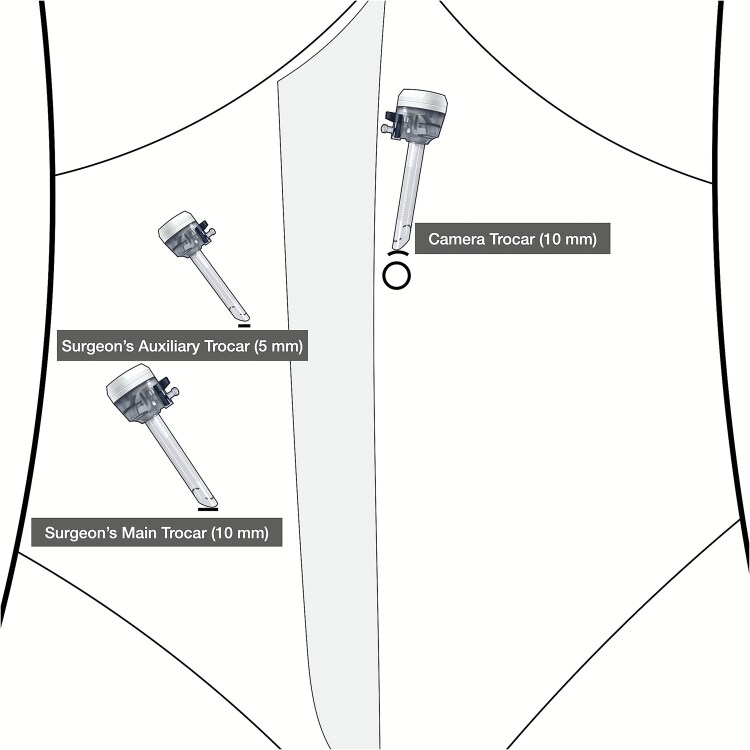
Trocar placement.

**Figure 2:**
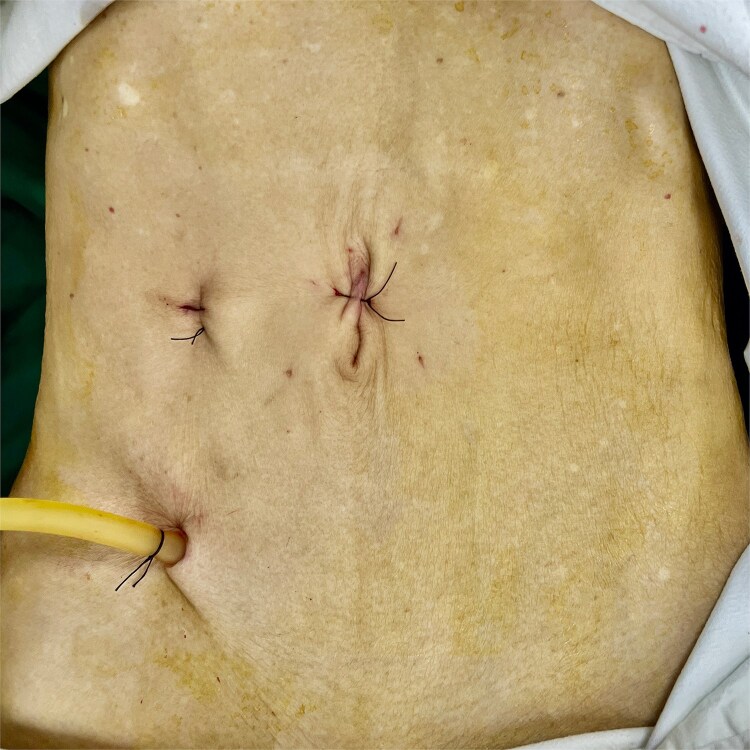
Postoperative abdominal wall.

A 72-year-old male with a BMI of 18.4 underwent a colonoscopy on 23 June 2025, which revealed a 5 cm adenocarcinoma in the sigmoid colon, staged cT3N0M0, with no metastases. He had no health issues, was considered fit for laparoscopic surgery, and underwent a NOSES IV procedure on 27 June 2025. The patient followed a clear liquid diet before surgery, underwent bowel preparation with polyethylene glycol, and received general anesthesia with intubation. He was positioned in lithotomy. A three-port trocar was used instead of five, with two 10 mm and one 5 mm trocars (Fig. 1). Aside from this modification (see Supplementary Video 1), other procedures adhered to the standard NOSES IV protocol. The operation lasted 90 minutes with a blood loss of 5 ml, requiring no conversion to laparotomy, and no abdominal wall stoma was created (Fig. 2). Postoperative therapy included ceftazidime (2 g) twice daily for four days. Ambulation was started on day 2, a liquid diet was resumed on day 3, and the patient was discharged on day 12 without any complications. Pathology showed a moderately differentiated adenocarcinoma of the sigmoid colon, with no cancer in the lymph nodes or margins, staged pT3N0M0.

### Technique (Figs 3 and 4)

**Figure 3:**
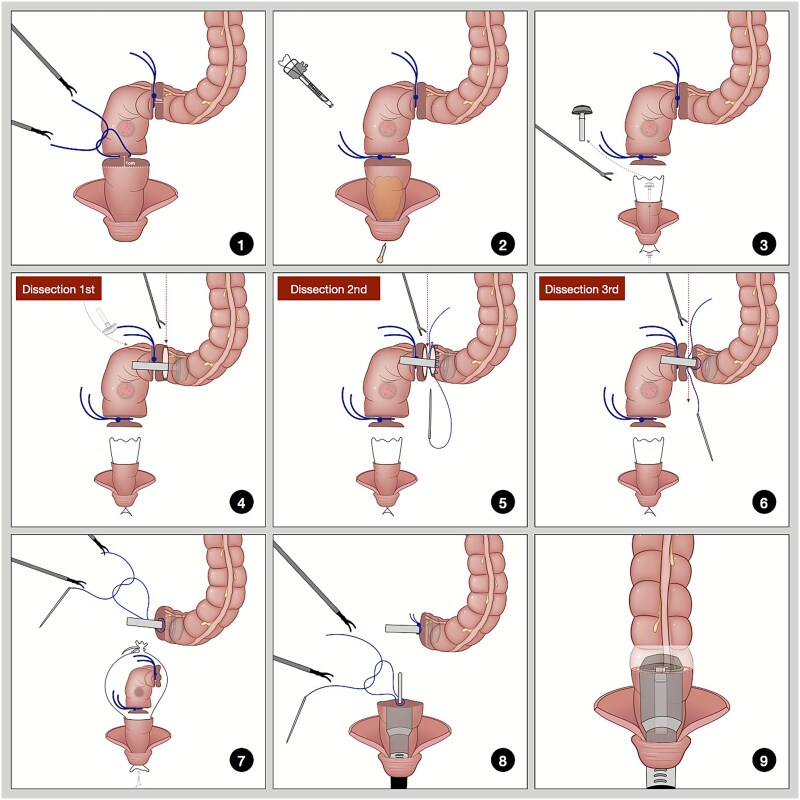
Illustration of three-step dissection with straight needle suturing.

**Figure 4:**
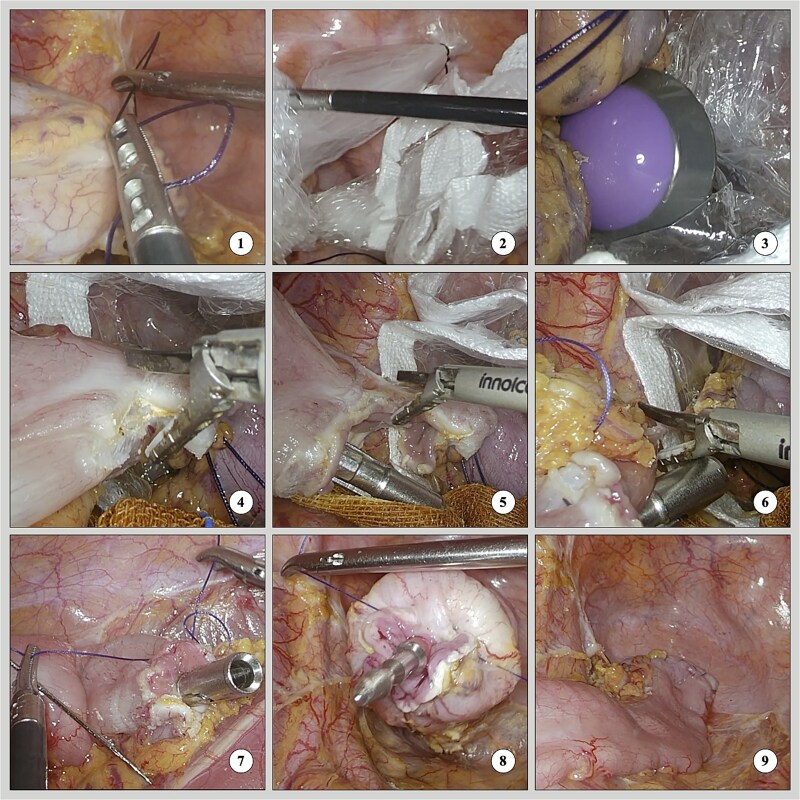
Intraoperative screenshots of three-step dissection with straight needle suturing.

Isolate and ligate the sigmoid colon and rectum 1 cm from the pre-cut line.
Insert a plastic sleeve through the trocar and disinfect the distal rectum.
Cut the rectum below the distal ligature with an ultrasonic scalpel, pull out the sleeve, and insert the anvil.
The anterior wall of the sigmoid colon was dissected above the proximal ligature, the opening was cleaned, and the posterior wall was used for traction while positioning the anvil.
Dissect most of the posterior wall of the sigmoid colon, then suture the anterior and posterior walls of the proximal sigmoid segment.
Continue dissecting the remaining posterior wall of the sigmoid colon.
Tighten the purse string to secure the anvil, place the specimen in the sleeve, and remove it through the anus.
Suture the rectal stump, insert the stapler, and secure the connecting rod.
Engage the anvil and rod, fire to complete the anastomosis, and check for leaks.

## Discussion

The use of NOSES is increasing and may become standard for small colorectal tumors [11]; however, inexperienced doctors might find manual suturing less advantageous [12]. Reduced-port laparoscopic surgery can limit visualization and increase operation time and complications. This report presents a three-step dissection technique using a straight needle for suturing the anvil to simplify intracorporeal colorectal anastomosis.

Surgery for upper rectal and sigmoid colon cancers involves inserting the anvil into the abdominal cavity through the anus. Placing an anvil during total laparoscopy is more difficult because of the need to secure the proximal bowel for sutures. Existing research on double-stapler technology [13, 14] indicates that it may increase training needs and reliance on consumables, leading to considerations of its role in assisting with colorectal anastomosis during early cases [15–17]. Currently, with the era of total laparoscopy, while the distal bowel part of the procedures is simpler, the focus has shifted to the difficulties of proximal bowel anvil placement. The initial step was to incise the anterior wall of the sigmoid colon and position it for self-traction with a transanal assistant to facilitate anvil insertion. Then, a large portion of the posterior wall was divided, easing suturing because of the fixed bowel segments. Finally, a straight needle was used to place a full-thickness horizontal mattress suture on the anterior wall and rotated for the posterior wall, enhancing control and precision. This modification allows securing the purse-string with two stitches, enhancing laparoscopic efficiency and decreasing operative time.

Additionally, the double ligation technique eliminates the need for a linear cutting closure device, thus reducing costs. Furthermore, the advanced surgical capabilities of the da Vinci robot allow for precise intestinal transection and purse-string suturing [18], along with evidence that robotic purse-string suturing can decrease surgery time for rectal cancer and reduce blood loss [19], and that robotic, end-to-end manual colorectal anastomosis is safe and feasible [20], this improvement is not only applicable to laparoscopic NOSES. It can also be used in robotic NOSES.

This report emphasizes the importance of proper anvil insertion and the selection of the right tubular stapler to avoid complications. Simultaneous specimen extraction and reconstruction may increase the risk of tumor contamination, highlighting the need for further studies to evaluate the safety and effectiveness of this modified approach.

## Conclusion

Three-step dissection with straight needle suturing simplifies anvil placement in intracorporeal colorectal anastomosis and may promote the adoption of NOSES.

## Supplementary Material

Three_Step_Dissection_with_Straight_Needle_Suturing_rjaf1008
